# Clustering and predicting VCA patient populations - a machine learning approach into vascularized composite allotransplantation

**DOI:** 10.3389/fimmu.2026.1894136

**Published:** 2026-08-19

**Authors:** Leonard Knoedler, Tobias Niederegger, Thomas Schaschinger, Carsten Stahl, Jule Brandt, Curtis L. Cetrulo, Max Heiland, Gabriel Hundeshagen, Jan O. Voss, Alexandre G. Lellouch

**Affiliations:** 1 Department of Oral and Maxillofacial Surgery, Corporate Member of Freie Universität Berlin and Humboldt-Universität zu Berlin, Charité–Universitätsmedizin Berlin, Berlin, Germany; 2 Division of Plastic and Reconstructive Surgery, Cedars Sinai Hospital, Los Angeles, CA, United States; 3 BG Klinik Ludwigshafen, Department of Hand, Plastic, and Reconstructive Surgery, Burn Center at Heidelberg University, Ludwigshafen, Germany; 4 Team Endotheliopathy and Hemostasis Disorders, Inserm The Paris Cardiovascular Research Center, Université Paris Cité, Paris, France; 5 Hematology Department, AP-HP, Hôpital Européen Georges Pompidou, Paris, France

**Keywords:** cluster analysis, machine learning, recipient characteristics, transplant types, vascularize composite allotransplantation (VCA)

## Abstract

**Background:**

Vascularized composite allotransplantation (VCA) enables functional and aesthetic restoration after devastating tissue loss. Yet, data-driven profiling of VCA recipients remains scarce. Identifying demographic and procedural patterns may improve transplant recipient selection, program planning, and perioperative care.

**Methods:**

VCA procedures in the Organ Procurement and Transplantation Network (OPTN, 1998–2023) were preprocessed, imputed, and clustered using weighted HDBSCAN. Resulting cluster patterns, temporal and geographic trends were analyzed. ARIMA models were applied to forecast patient demographics.

**Results:**

Among 107 recipients (mean age 37 ± 12 years; BMI 25 ± 5 kg/m²; 57% female), clustering analyses confirmed gender and VCA type as the primary differentiating dimensions, with face (19%) and upper-limb transplants (30%) predominantly performed in men and uterus transplants (31%) concentrated in United Network for Organ Sharing (UNOS) Regions 4 and 10. The facial VCA subgroup clustered regionally in Regions 1 and 9 (n = 13) and was characterized by comparatively high recipient age (42 ± 11 years) and long waiting times (246 ± 198 days). In the balanced clustering solution, 42% of recipients were classified as noise, highlighting substantial population heterogeneity. Temporal analyses showed declining activity in abdominal wall (11%) and upper-limb procedures, whereas ARIMA forecasts indicated stable age and BMI distributions through 2027.

**Conclusions:**

This study provides the first machine-learning–based characterization of the national VCA recipient population using OPTN data. Rather than discovering entirely new recipient categories, the clustering framework successfully reproduced established population structures while identifying secondary regional and temporal patterns. Furthermore, density-based clustering highlighted substantial recipient heterogeneity through explicit outlier detection, thereby potentially providing a scalable framework for future registry-based analyses that incorporate immunological, outcome-related, and more granular clinical variables to enable earlier identification of atypical recipients and more targeted program planning.

## 1 Introduction

Over 500 million amputees worldwide and over 5 million severe burns annually underline the pressing need for advanced reconstructive surgery (1, 2). Conventional reconstructive surgery often fails to restore both form and function. Vascularized composite allotransplantation (VCA) aims to achieve functional recovery and aesthetic reconstruction in individuals with extensive and irreversible tissue defects (3, 4). VCA surgery describes the transfer of a composite graft containing multiple tissues and is considered life-enhancing rather than life-saving (5, 6).

Over the past two decades, more than 50 facial, 100 uterine, 40 abdominal wall and 150 hand VCAs have been performed (7–10). Beyond restoring essential functions, VCA has been demonstrated to help patients reclaim personal identity, facial expressiveness, social belonging and independence (11–13). In upper-extremity VCA, 91% and 82% of recipients have been shown to develop tactile sensibility and regain partial discriminative sensibility (14). In face VCA, the first 50 cases have shown a promising 5-year-survival rate of 85% (7). Further, an AI-based analysis of 14 face VCA recipients demonstrated favorable aesthetic outcomes across age, ethnicity, and gender groups (15). However, persisting hurdles challenge the widespread clinical adoption of VCA. Lifelong immunosuppression has been implicated with carcinogenic and metabolic side effects, whereas chronic rejection can lead to irreversible graft deterioration, resulting in 2 facial retransplants (16–19). Moreover, high perioperative costs and frequent postoperative follow-ups are considered additional hurdles (15, 20, 21).

Analyzing patient populations may optimize perioperative care, yet current evidence remains largely descriptive, with limited application of advanced analytical methods to national VCA registry data. This research gap leaves untapped potential to better characterize VCA patient populations, identify atypical recipient profiles, improve understanding of recipient diversity, and support future transplantation planning.

In this study, we analyzed the current VCA patient population within the Organ Procurement and Transplantation Network (OPTN) and applied machine-learning techniques to evaluate recipient population structure (20, 21). The goal of our work was to determine whether unsupervised clustering could recover known population structures, identify atypical recipient profiles and secondary organizational patterns, and forecast future demographic trends within the national VCA population. Overall, this approach provides a scalable analytical framework that may support more targeted VCA recipient screening, early identification of atypical recipients requiring individualized care pathways, and evidence-based resource allocation for future VCA program development.

## 2 Methods

### 2.1 Data source and patient selection

We analyzed VCA transplant and follow-up data from OPTN Standard Transplant Analysis and Research (STAR) files, including all VCA types performed in the United States between 1998 and 2023.

An eight-year-old bilateral upper-limb recipient, a pediatric outlier, as well as all larynx and trachea transplants due to insufficient case numbers and one duplicate record, were excluded from the final analytical cohort.

### 2.2 Variable extraction

We preprocessed the VCA dataset to enable meaningful characterization of recipient population structure, prioritizing transformations that enhanced comparability of medical, demographic, and procedural characteristics. Each patient was mapped into a high-dimensional Euclidean space in which inter-patient distances reflected underlying similarities.

**Figure 1:**
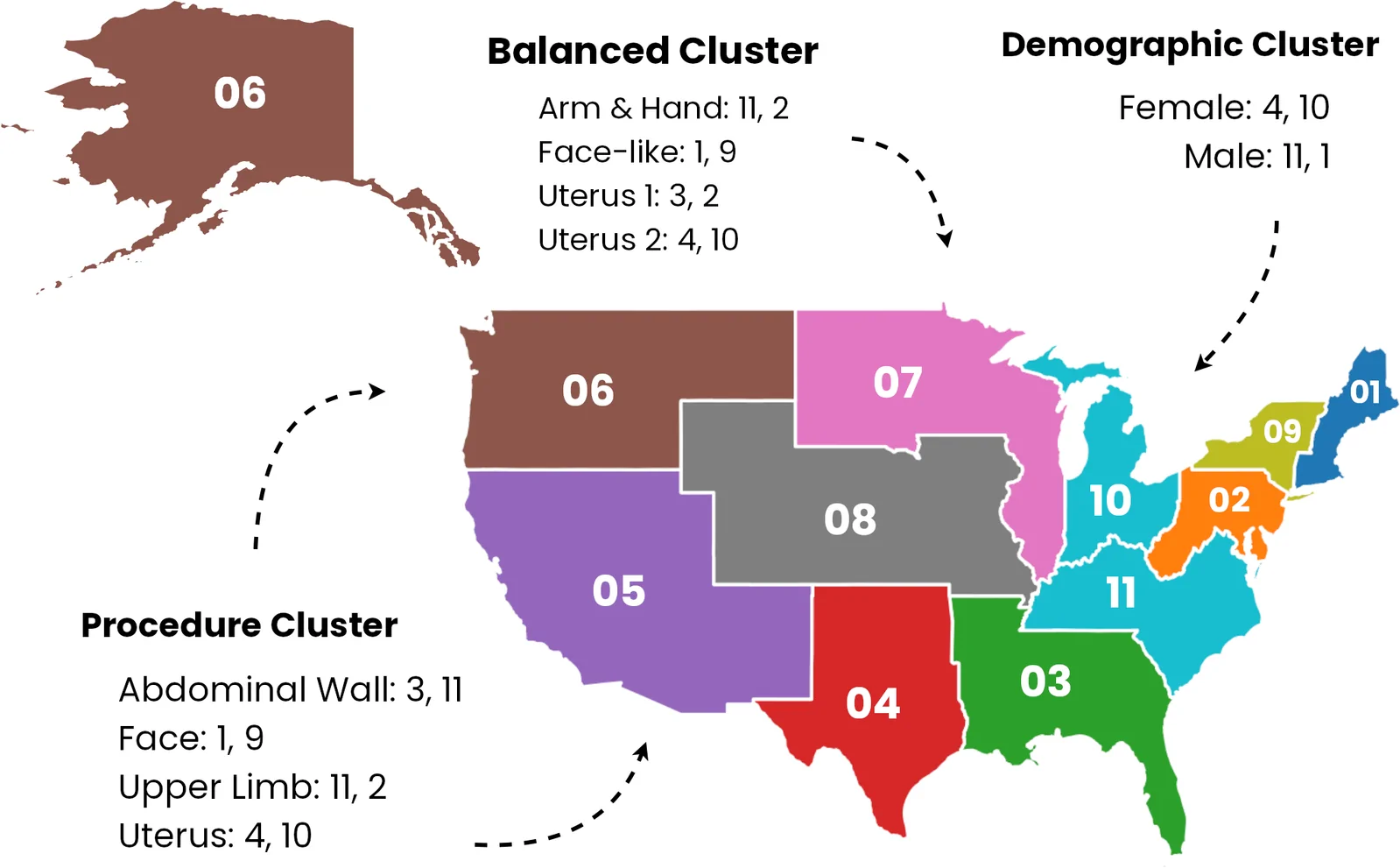
UNOS Regions of OTPN (Source: Regions - OPTN).

Demographic characteristics included gender, age (years), and ethnicity; medical characteristics comprised body mass index (BMI, weight [kilograms]/(height [meters])²), ABO blood type, waiting time (days) between registration and transplant; and procedural characteristics such as the United Network for Organ Sharing (UNOS) Region in which the procedure occurred. The UNOS divides the U.S. into 11 geographic regions (**Figure 1**).

Missing data were handled using targeted imputation strategies. Numerical values (e.g. BMI (n = 12, 11% missing), waiting time (n = 35, 32% missing)) were imputed using the median value for each respective transplant type.

To prevent the algorithm from artificially grouping patients based on shared missing data rather than true clinical similarity, records with missing blood types (n = 26, 24%) were assigned to a distinct ‘missing’ category. Crucially, the distance metric was modified so that a ‘missing’ value was defined as equidistant from all other categories, including other ‘missing’ values. This ensures that the absence of data does not erroneously act as a shared phenotypic trait.

Categorical variables were numerically encoded (**Supplementary Table 1**). A procedural risk score was derived from a logistic regression model predicting adverse outcomes based on donor–recipient compatibility and ischemia time, providing a continuous measure of procedural complexity (**Supplementary Material** Numerical Representations). UNOS Regions were mapped to representative geographic centroids to approximate inter-regional distances.

### 2.3 Statistical analysis and machine learning

The analysis combined unsupervised learning through clustering with statistical modeling of temporal dependencies. Clustering was used to characterize recipient population structure beyond simple transplant-type stratification, while time-series analysis assessed BMI and age trajectories. All analyses were performed using Python 3.13 in a local Jupyter Notebook. Data manipulation was performed with pandas 2.3.2, clustering with scikit-learn 1.7.1 (22), and fitting with ARIMA (p, d, q) models in statsmodels 0.14.4 (23).

To characterize recipient population structure, a custom distance function was implemented that assigned adjustable weights to demographic, medical, and procedural variables (see **Supplementary Material** Distance Computation, **Supplementary Table 2**). This design enabled flexible emphasis on specific patient characteristics and allowed the generation of cluster solutions highlighting demographic, medical, procedural, and overall commonalities.

Several clustering algorithms were tested. HDBSCAN (24) was selected for its robustness and ability to detect clusters of varying density and shape while identifying noise and substructures within otherwise homogeneous groups, such as patients associated with the same procedure (see Uterus I and II in Results). Importantly, unlike many conventional clustering approaches, HDBSCAN explicitly labels observations that do not belong to any stable cluster, enabling identification of atypical recipient profiles. Using the custom distance function, three main configurations - demographic-focused, procedure-focused, and balanced clusters - were generated, serving as the foundation for all subsequent descriptive and temporal analyses.

To validate the clusters and assess their overall robustness, we evaluated the mean HDBSCAN membership probability. This metric assigns a membership score based on spatial density: VCA patients at the core of a cluster receive high probabilities, whereas peripheral cases receive lower scores. Consequently, the mean membership probability across all patients in a group serves as a quantitative measure of cluster stability.

Temporal developments were assessed by analyzing the distribution of transplant dates within each cluster. For each cluster, the temporal distribution of transplants was visualized using boxplots of transplant year, providing an exploratory approach for assessment of temporal patterns without relying on model-based assumptions unsuitable for sparse data.

Due to sparse data an aggregated ARIMA model was applied to forecast the BMI and age for all patients. An ARIMA (25) model describes how past observations and random fluctuations relate to one another, removes long-term trends through differencing (integration), and uses these patterns to forecast future values. Stationary was evaluated using the Augmented Dickey–Fuller test (26). The appropriate model order was determined based on the partial autocorrelation (PACF) and autocorrelation (ACF) functions.

This study was conducted and reported in accordance with the STROBE guidelines.

## 3 Results

### 3.1 Cluster demographics

Overall, n = 107 (100%) patients were analyzed. Patient age ranged from 19 to 69 years (mean ± SD of 37 ± 12), of whom n = 61 (57%) were female. Their BMI ranged from 17.7 to 43.9 kg/m² with a mean ± SD of 25 ±5. Across all clustering configurations, recipient sex and VCA transplant type represented the strongest determinants of population structure. Notably, upper-limb and face transplants were predominantly carried out in male recipients (face: n=15, 75%, upper-limb: n = 22, 66%) (**Table 1**).

**Table 1:** Patient demographics after cluster.

Cluster-types	Subgroups	Sample size	Mean (± SD) age (years)	Female	Mean (± SD) BMI (kg/m²)	Mean (± SD) waiting time (days)	Top 2 UNOS regions	Top 2 VCA types	Mean cluster probability/robustness
Demographic	Female Cohort	57	32.37(± 7.76)	n=57, 100%	24.72(± 3.56)	165.07(± 231.00)	4 (n=25, 44%), 10 (n=10, 18%)	Uterus (n=40, 70%), Upper Limb Bilateral (n=6, 11%)	55%
	Male Cohort	42	39.95(± 11.85)	n=0, 0.00%	26.70(± 5.91)	240.83(± 224.42)	11 (n=11, 26%), 1 (n=10, 24%)	Face (n=12, 29%), Upper Limb Bilateral (n=8, 19%)	90%
Procedure	Abdominal Wall	12	31.83(± 10.74)	n=4, 33%	24.47(± 2.42)	105.42(± 52.14)	3 (n=10, 83%), 11 (n=1, 8.3%)	Abdominal Wall (n=12, 100%)	100%
	Face Transplants	20	41.75(± 13.83)	n=5, 25%	25.80(± 6.47)	279.40(± 111.22)	1 (n=10, 50%), 9 (n=4, 20%)	Face (n=18, 90%), Scalp (n=1, 5.0%)	90%
	Upper Limb Transplantations	32	42.19(± 13.25)	n=11, 34%	26.33(± 5.42)	303.34(± 324.46)	11 (n=13, 41%), 2 (n=9, 28%)	Upper Limb Bilateral (n=16, 50%), Upper Limb Right (n=8, 25%)	95%
	Uterus	33	31.52(± 4.84)	n=33, 100%	24.62(± 3.51)	92.30(± 152.37)	4 (n=25, 76%), 10 (n=8, 24%)	Uterus (n=33, 100%)	75%
Balanced	Arm & Hand group	10	43.90(± 8.89)	n=0, 0.00%	28.38(± 3.83)	93.95(± 143.14)	11 (n=9, 90%), 2 (n=1, 10%)	Upper Limb Right (n=5, 50%), Upper Limb Left (n=4, 40%)	98%
	Face-like subgroup	15	38.87(± 13.52)	n=2, 13%	25.61(± 5.26)	257.13(± 107.14)	1 (n=6, 40%), 9 (n=4, 27%)	Face (n=13, 87%), Scalp (n=1, 6.7%)	87%
	Uterus I	6	31.17(± 4.22)	n=6, 100%	24.09(± 4.54)	130.50(± 90.04)	3 (n=4, 67%), 2 (n=2, 33%)	Uterus (n=6, 100%)	100%
	Uterus II	31	31.74(± 4.45)	n=31, 100%	24.52(± 3.59)	78.10(± 124.35)	4 (n=24, 77%), 10 (n=7, 23%)	Uterus (n=31, 100%)	92%
	Noise	44	39.68(± 14.76)	n=22, 50%	25.77(± 5.56)	302.71(285.71)	3(n=12, 27%), 1 (n=10, 22%)	Upper Limb Bilateral (n=15, 34%)	0%

#### 3.1.1 Balanced clusters

In this cluster, continuous and categorical features from all patients (n = 62, 58%) contributed equally, with n = 45 (42%) patients being classified as noise due to the requirement for similarity across all major feature types (demographic, medical, and procedural). The robustness and temporal distribution of the noise cases reveal no underlying structure within the noise cluster; rather, this lack of structure reflects true patient heterogeneity across the full set of characterizing variables.

The Arm & Hand cluster (n = 10, 9%) represented a male only group with the highest mean BMI of 28.4 ± 3.8 kg/m², showing a concentration in UNOS Region 11 (n = 9, 90%). The Face-like subgroup similarly consisted predominantly of male recipients (n = 13, 87%) and exhibited waiting times (257.1 ± 107.1 days) tied to UNOS Regions 1 (n = 6, 40%) and 9 (n = 4, 27%). Furthermore, two subgroups of uterus transplant recipients emerged—Uterus I and Uterus II—sharing the same transplant type, differing by UNOS Regions, waiting time, and blood type. Uterus II, in particular showed a shorter mean waiting time of 78.1 ± 124.4 days compared to the Face-like subgroup, included recipients with mixed blood types (Uterus I contained exclusively patients with blood type A), and stronger regional concentration in UNOS Regions 4 (n = 24, 77%) and 10 (n = 7, 23%) (**Table 1**).

#### 3.1.2 Demographic-focused clusters

Giving more weight to the demographic features resulted in two clusters, with one being exclusively male (n = 42, 40%) and one exclusively female (n = 57, 53%). The female cohort was younger than the male cohort, averaging 32.4 ± 7.8 years compared with 40.0 ± 11.9 years, respectively. It also exhibited a lower mean BMI (24.7 ± 3.6 kg/m² vs. 26.7 ± 5.9 kg/m²) and shorter average waiting times (165.1 ± 231.0 days vs. 240.8 ± 224.4 days). Most female cases were concentrated in UNOS Regions 4 (n = 25, 44%) and 10 (n = 10, 18%), dominated by uterus transplants (n = 40, 70%), whereas the male cohort was more heterogeneous, combining face (n = 12, 29%) and bilateral upper-limb (n = 8, 19%) procedures, primarily in UNOS Regions 11 (n = 11, 26%) and 1 (n = 10, 24%) (**Table 1**).

#### 3.1.3 Procedure-focused clusters

By emphasizing VCA type and UNOS Region, four procedural clusters emerged: abdominal wall, facial, upper-limb, and uterus transplants. Abdominal wall transplants (n = 12, 11%) showed a strong geographic concentration in UNOS Region 3 (n = 10, 83%) and a comparatively balanced gender distribution (n = 4, 33% female). The face transplant cluster (n = 20, 19%) included the oldest recipients (41.8 ± 13.8 years) and longest mean waiting times (279.4 ± 111.2 days), mostly located in UNOS Regions 1 (n = 10, 50%) and 9 (n = 4, 20%). Upper-limb transplant cluster (n = 32, 30%) showed the highest mean BMI (26.3 ± 5.4 kg/m²) and the broadest demographic variation, with the second highest standard deviation in BMI and Age (± 13.25 years) and the most balanced gender distribution (n = 11, 34% female). Uterus transplant cluster (n = 33, 31%) was concentrated in UNOS Regions 4 (n = 25, 76%) and 10 (n = 8, 24%) and exhibited a shorter average waiting time (92.3 ± 152.4 days) (**Table 1**).

### 3.2 Temporal distribution of clusters

The timeline of all transplants extends from January 1999 to May 2023, covering cases only from the first half of 2023.

Within the balanced cluster, Uterus I included procedures predominantly performed between November 2018 and November 2022 with n = 4 (67%) procedures in the UNOS Region 3, whereas procedures occurring in the time period of February 2016 to March 2022 were most commonly performed in UNOS Regions 10 and 4. Male patients with elevated BMI undergoing arm or hand transplantation in UNOS Region 11 (n = 9, 90%) increased from 2000 to 2010, followed by a gradual decline (n=4, 40% from 2011 to 2019) with the last two transplants being three years apart.

**Figure 2:**
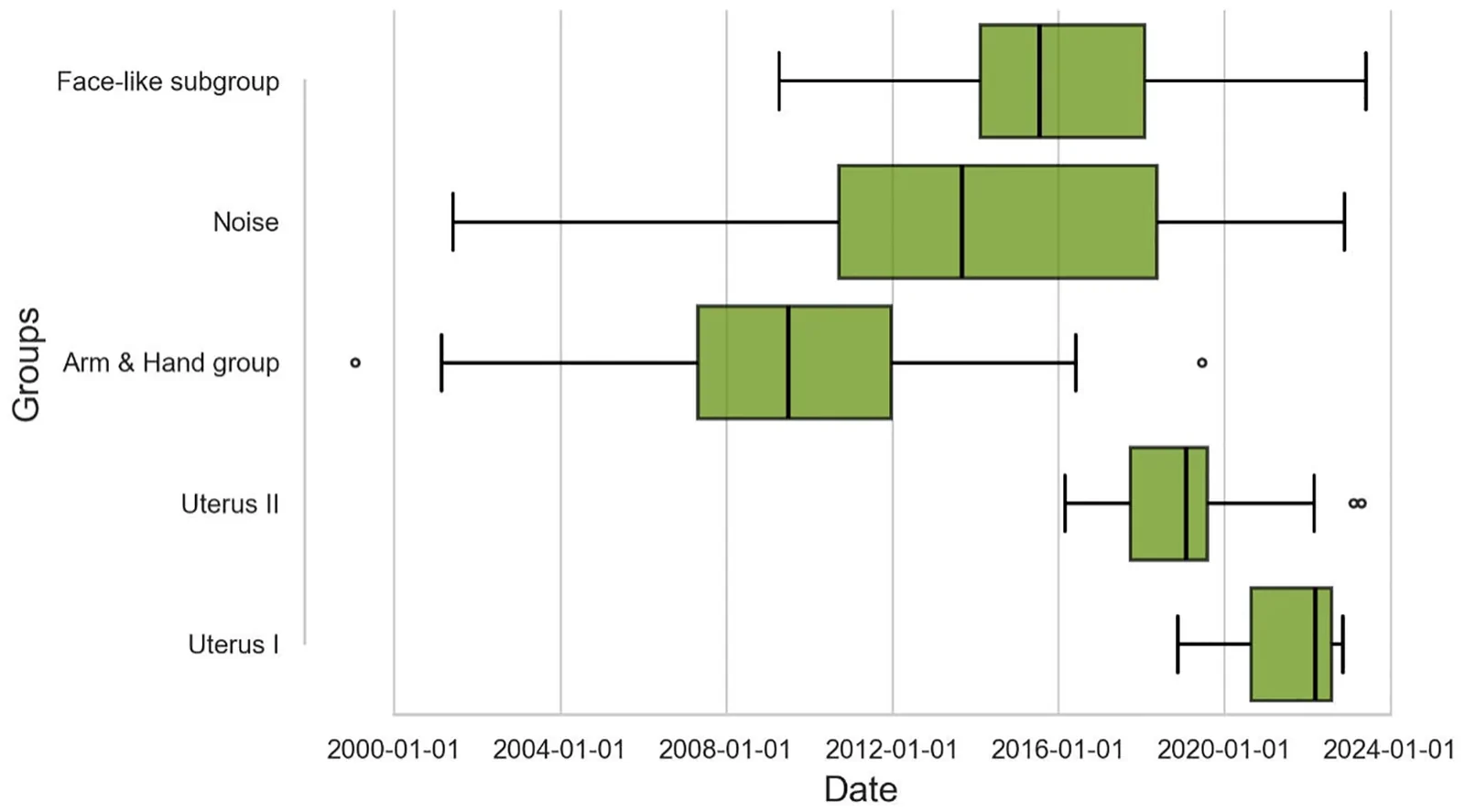
Boxplot of transplant times for balanced clusters.

**Figure 3:**
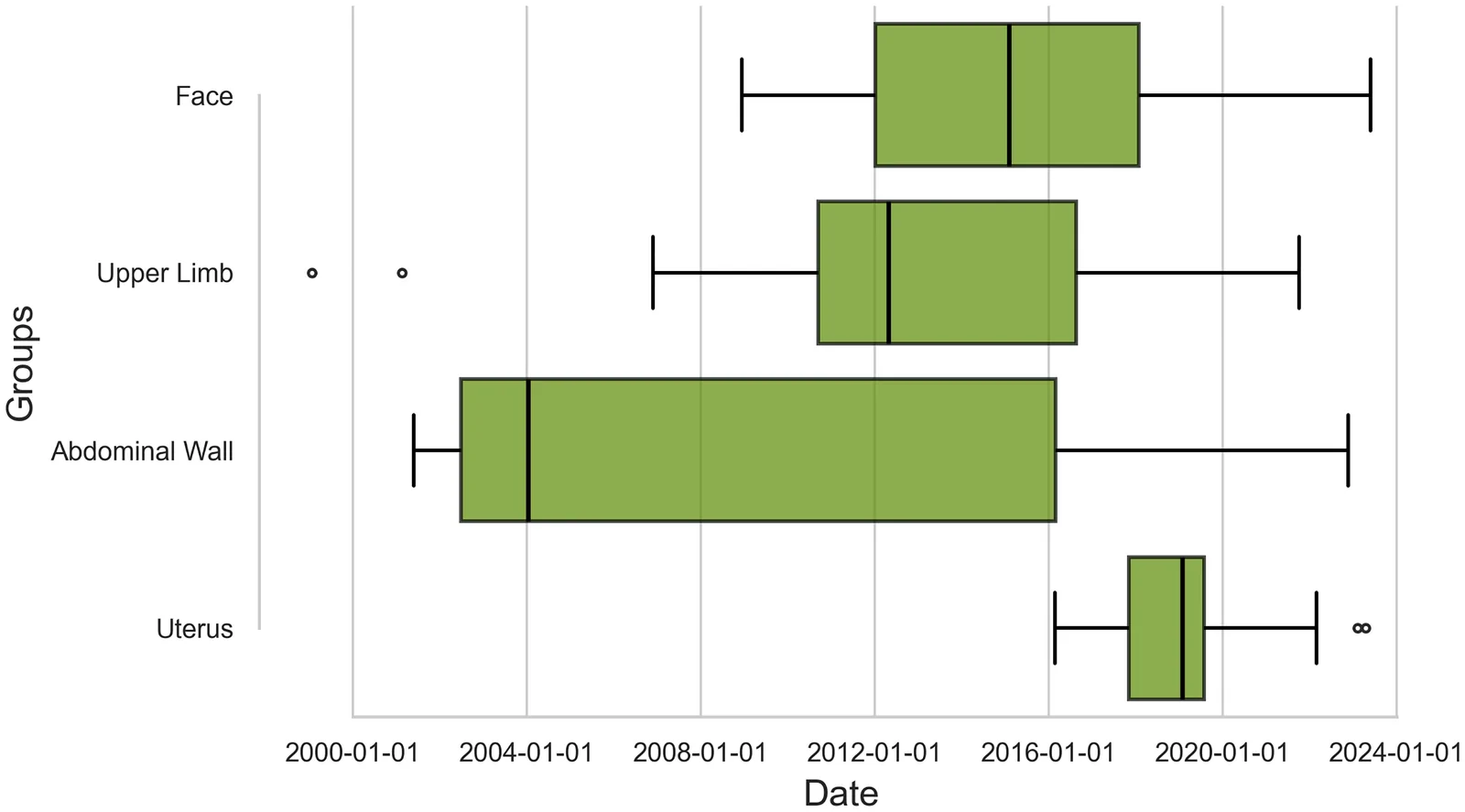
Boxplot of transplant times for procedure clusters.

Within the procedure-focused clusters, in the period from 2009 to 2023, n = 14 (70%) face transplant procedures were performed in UNOS Regions 1 and 9. One of the three procedures performed in 2023 involved a face transplant, while the remaining two were uterus transplants. These represent the most recent transplant types recorded in the dataset. Abdominal wall transplants showed the highest procedural frequency (n = 7, 58%) between 2002 and 2004, followed by a gradual decline, with n = 5 (42%) procedures performed from 2005 to 2022 (**Table 1**; **Figures 2**, **3**).

### 3.3 BMI and age forecast

**Figure 4:**
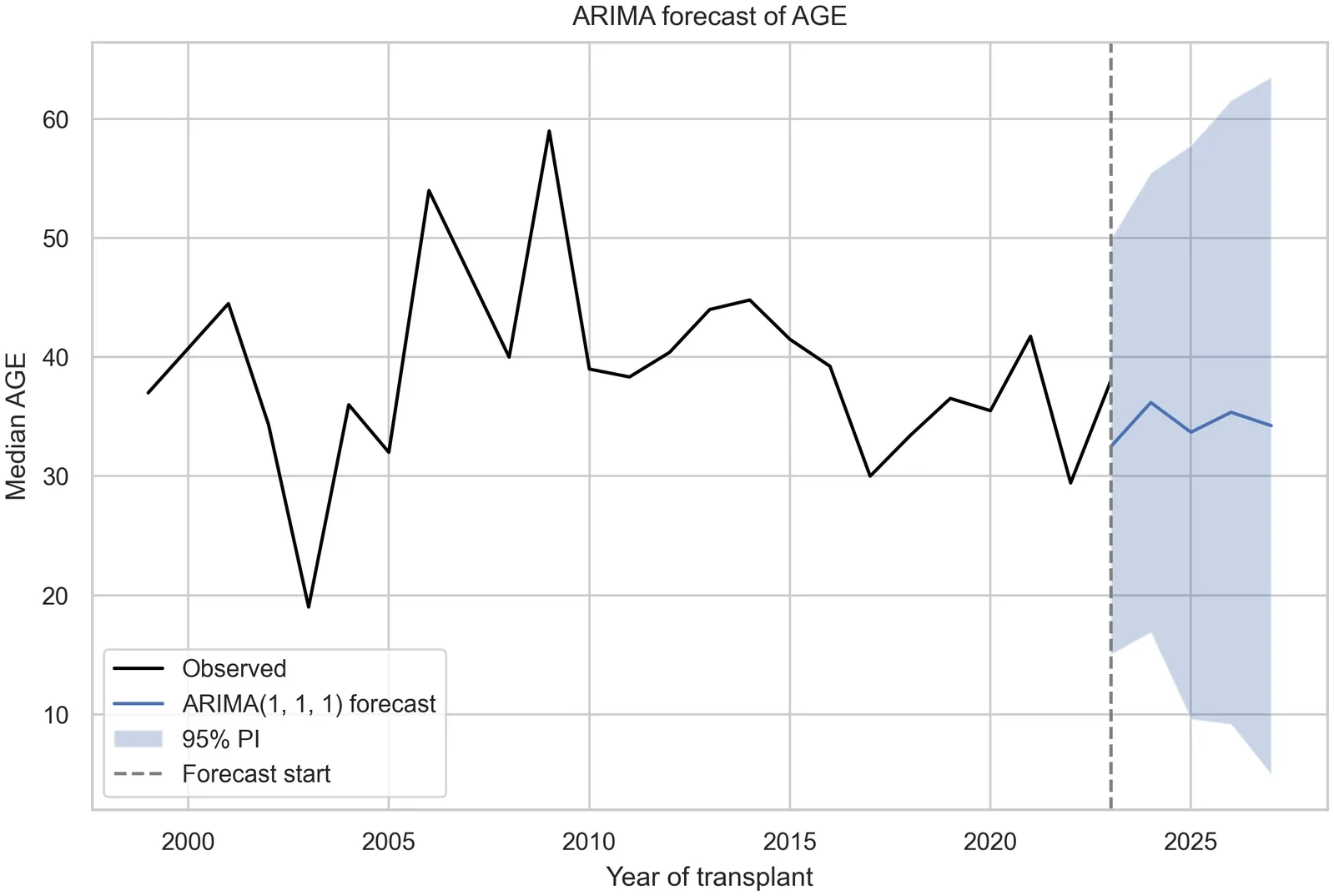
ARIMA (1, 1, 1) Forecast of age for all VCA-Patients with projections extending to 2027.

**Figure 5:**
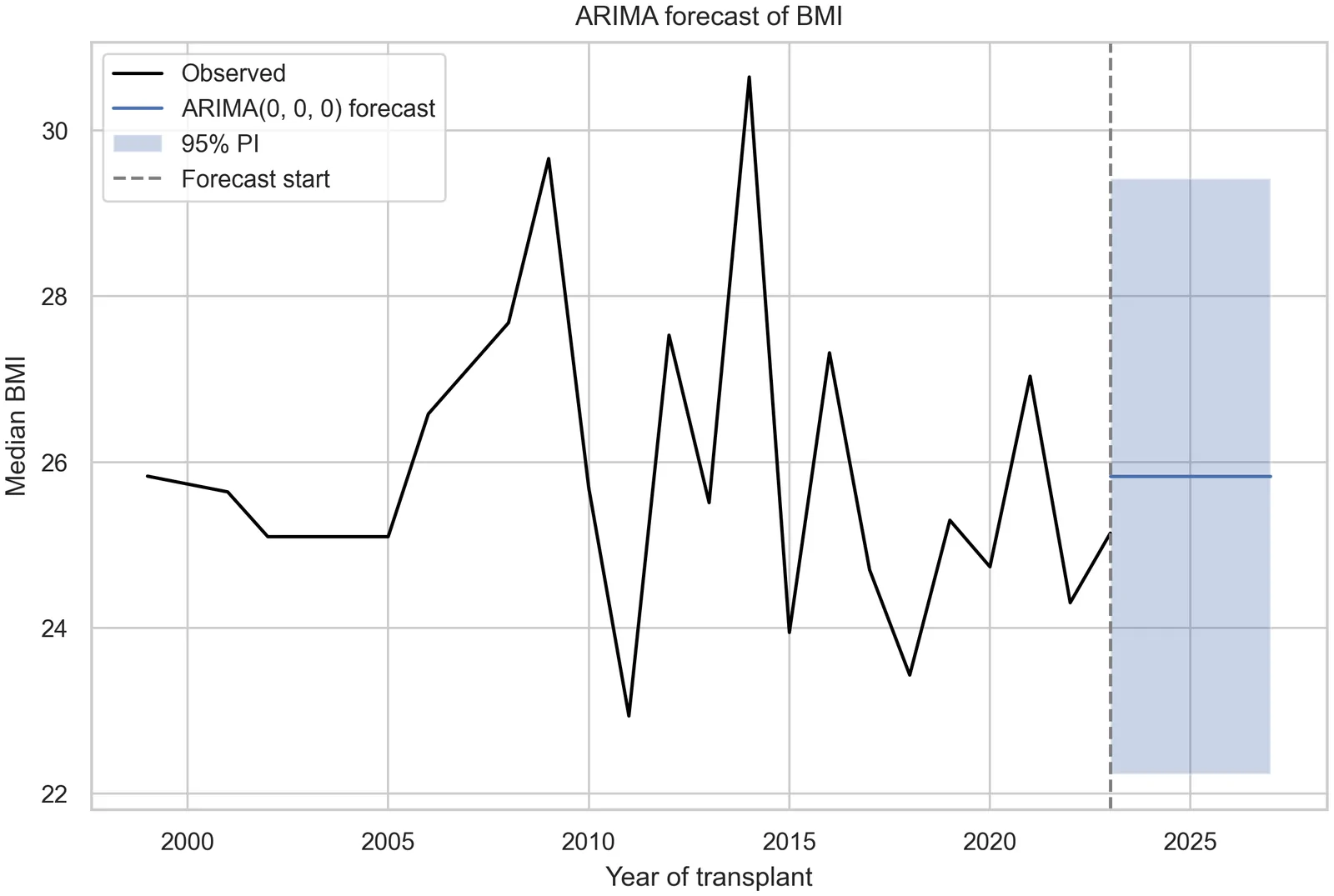
ARIMA (0, 0, 0) Forecast for BMI of all VCA Patients with projections extending to 2027.

The BMI series showed no significant serial correlation, fluctuating around a mean of 25 kg/m². An ARIMA (0, 0, 0) (=(p, d, q)) constant model was appropriate. The age series required differencing to achieve stationarity and showed weak autoregressive and moving-average components, resulting in an ARIMA (1, 1, 1) model reflecting short-term variability within an overall stable trend. Based on these projections, BMI is expected to remain around 25–26 kg/m², while mean recipient age will likely persist in the mid-thirties, showing only minor short-term fluctuations. Despite its integrated structure (d = 1), the model suggests minor short-term variation but no persistent temporal change. (**Figures 4**, **5**).

## 4 Discussion

This study represents the first application of machine-learning–based clustering to systematically characterize the VCA recipient population using a national multi-center registry (9, 21, 27). Despite the growing number and complexity of VCA procedures, recipient populations have primarily been described using conventional epidemiologic approaches (28–30). Given the unique surgical, psychosocial, and ethical challenges inherent to VCA, such characterization is critical to provide tailored, patient-centered care and improve outcomes (31). VCA is often the final reconstructive option for patients with devastating tissue loss, where conventional techniques fail to restore both form and function, resulting in profound psychosocial burden, limitations in daily life, and significantly reduced quality of life (32–34). Traditional forecasting approaches, such as linear trend extrapolation, often fail due to the limited sample size, high variability, and irregular timing of VCA procedures, which violate the assumptions underlying these models. By establishing a scalable, machine-learning–based analytic framework, this study demonstrates how even a relatively small dataset can be systematically evaluated to characterize population structure and identify secondary demographic, procedural, geographic, and temporal patterns, while creating the foundation for future expansion as the OPTN VCA registry grows. Such an approach underscores the need to enrich the OPTN database with more granular, systematically collected data to unlock the full potential of AI-driven analyses. Rather than focusing on the discovery of entirely new recipient categories, clustering provides a complementary framework for evaluating whether known population structures can be recovered and whether additional organizational patterns exist within the national VCA population. By characterizing recipient population structure and highlighting areas of heterogeneity, this work aims to establish a methodological foundation for future registry-based analyses, patient stratification, and resource allocation in this evolving field (35).

Our clustering analysis confirmed that recipient sex and VCA transplant type represent the principal determinants of population structure within the contemporary U.S. VCA landscape. While these findings are consistent with established epidemiologic observations, the unsupervised framework additionally identified secondary geographic and procedural patterns, including the regional concentration of uterus transplantation in UNOS Regions 4 and 10 and the predominance of facial transplantation in Regions 1 and 9, where recipients were generally older and experienced longer waiting times. Temporal analyses further demonstrated stable recipient age and BMI distributions over time, suggesting limited demographic drift within the current VCA population.

Importantly, these results should not be interpreted as the discovery of previously unrecognized clinical entities. Rather, they demonstrate that an unsupervised clustering framework can recover known population structures without predefined labels while simultaneously identifying secondary organizational patterns within the dataset. The validity of this demonstration is further substantiated by robust assignment metrics, with high mean cluster probabilities confirming the structural stability of the groupings. Furthermore, 42% of recipients were classified as noise in the balanced clustering solution, reflecting substantial heterogeneity within the contemporary VCA population. This highlights a unique feature of density-based clustering approaches: the ability to identify recipients who do not conform to dominant demographic, procedural, and medical patterns. As larger transplant registries become available, such outlier detection may support the identification of unusual recipient characteristics, rare clinical scenarios, and potential access disparities warranting further investigation.

The broader literature on VCA underscores the importance of defining recipient populations to optimize outcomes, yet comprehensive demographic and procedural analyses remain limited. In particular, facial VCA has consistently been associated with long waiting times. One contributing factor is the scarcity of well-matched donors, exacerbated by challenging skin color-matching and limited public and professional awareness of facial transplantation as a treatment option (5, 36–38). Increased awareness, broader education efforts, and improved documentation of potential recipient pools could facilitate earlier identification of eligible patients and donors, helping to shorten wait times and ensure timely intervention. Moreover, continued investment in ethical frameworks, legal policy development, and cross-institutional registries is crucial to enable equitable access and overcome remaining hurdles to wider implementation (39). Recent work emphasizes that tailored perioperative and psychosocial support, guided by a better understanding of patient characteristics, is essential for both short-term surgical success and long-term functional reintegration (40–44). In this context, clustering via advanced computational methods offers a promising tool to characterize population structure and identify secondary demographic, procedural, and geographic relationships beyond conventional descriptive analyses. Such population-level characterization may support future efforts to align resources, counseling, and rehabilitation strategies with the needs of different recipient populations (21, 45). The value of personalized models has been echoed in various studies that show better adherence, psychological adjustment, and graft survival when support strategies are aligned with patient profiles (46, 47).

In parallel, forecasting future VCA case loads is critical to guide the development of dedicated VCA programs and ensure efficient resource allocation. Previous studies have highlighted the high cost and complexity of VCA procedures, which place a significant burden on institutional logistics and long-term follow-up systems. Reliable prediction models would help providers proactively plan for procedural volume, improve surgical team readiness, and prevent bottlenecks in donor–recipient matching (11, 12, 48–50). Furthermore, predicting future VCA patient characteristics, including age and BMI trends, offers a pathway toward more individualized risk assessment and tailored preoperative health optimization, which is increasingly recognized as a determinant of successful postoperative outcomes (48, 51). Taken together, this body of literature supports a shift toward data-driven frameworks for VCA planning and execution. Advancing our understanding of patient pools, especially in high-need, high-complexity areas like facial transplantation, will be key to reducing access delays, enhancing outcome sustainability, and guiding the next phase of ethical and clinical evolution in the field.

For patients, this study could represent an important step toward more personalized access to complex reconstructive procedures like facial or limb transplantation. By characterizing recipient population structure and forecasting future trends, this research may contribute to improved program planning, donor–recipient matching, and allocation of clinical resources. For physicians and healthcare teams, these insights could assist in anticipating future caseloads, guiding the development of specialized VCA programs, and informing more efficient distribution of surgical, financial, and support resources. As tailored pre- and postoperative care might improve both immediate outcomes and long-term quality of life, future research will be essential to validate these findings, refine predictive tools, and expand our understanding of the evolving VCA recipient population.

## 5 Limitations

This study has several limitations. First, although the OPTN STAR database provides the most comprehensive national registry for VCA, the small sample size and incomplete follow-up data limit the generalizability of our findings. Many entries lack consistent longitudinal information, constraining the ability to assess outcomes or temporal dynamics. Second, imputation of missing values, while carefully performed, introduces potential bias, especially for variables with high heterogeneity such as waiting time or BMI. Third, the clustering and time-series analyses are exploratory in nature and depend on the specific distance metrics and parameter settings chosen; alternative weighting schemes might yield different patient groupings. Fourth, regional trends may reflect reporting or institutional biases rather than true procedural shifts. In addition, at the time of the study analysis, the OPTN STAR database did not yet include data later than 08/2023. Furthermore, the OPTN dataset did not contain sufficiently detailed immunological variables, including donor-specific antibodies, sensitization status, immunosuppressive regimens, rejection episodes, or comprehensive HLA matching data. Consequently, the present study should be interpreted as a population-structure analysis rather than an immunologic risk-modeling study. Further, while regression parameters were successfully validated across several VCA types, formal validation metrics could not be computed for subgroups exhibiting zero outcome variance. Lastly, as VCA remains a rare and evolving field, results should be interpreted cautiously until larger, standardized datasets with broader clinical and psychosocial variables become available for validation.

## 6 Conclusion

In conclusion, this study represents the first machine-learning–based characterization of the national VCA recipient population using OPTN registry data. Rather than discovering entirely new recipient categories, the clustering framework successfully reproduced established population structures while identifying secondary regional and temporal patterns within the contemporary U.S. VCA landscape. The balanced clustering solution further highlighted substantial recipient heterogeneity through explicit outlier detection, identifying patients whose multivariate profiles deviate from established population norms and who may therefore warrant individualized perioperative risk assessment, closer immunological monitoring, or investigation of systemic access disparities. As larger registries incorporate more detailed clinical, immunological, and outcome-related variables, this framework may provide a scalable foundation for future population-level analyses and program planning in VCA.

## Data Availability

Publicly available datasets were analyzed in this study. This data can be found here: OPTN.
