# Supplementary material for: Clustering and predicting VCA patient populations - a machine learning approach into vascularized composite allotransplantation
Source: Front Immunol. 2026 Aug 19;17:1894136. doi: 10.3389/fimmu.2026.1894136 (PMC13533772; doi:10.3389/fimmu.2026.1894136)
Supplement: Supplementary file 1 [file SupplementaryFile1.docx]

**Clustering and Predicting VCA Patient Populations: Supplementary Digital Content**

**-**

**A Machine Learning Approach into Vascularized Composite Allotransplantation**

**Numerical Representations**

Table 1 presents the numerical encoding of all variables used in the analysis. The level of complexity varies notably across features, ranging from simple dummy variables for categorical attributes such as *gender* and *ethnicity* to more complex composite scores that capture multiple dimensions of a feature, such as *VCA type*. To calculate the risk score using regression modeling, we stratified patients according to their VCA type and fitted an individual logistic regression model to each subgroup. The model equation can be written as follows:

$$p_{i}=\frac{1}{1+exp(-(\beta_{p,0}+\sum_{i=1}^{5} \beta_{p,j}x_{j}))}$$

Here $p_{i}$ describes the probability of a nonsurgical complication for the $i$th patient, while $\beta_{p,j}$ describes the parameter of the jth exogenous variable (j=1=Hemoglobin A1C,j=2=Serum Creatinine, j=3=ABO mismatch, j=3=ischemia times, and j=4=crossmatch results) of the $p$th VCA type. The parameter estimate  $\hat{\beta}_{p}=(\beta_{p,0},\ldots.\beta_{p,5})$ was then used as part of the VCA types numerical representation.

**Distance Computation**

To construct both data-driven and custom patient profiles that incorporate medical (e.g., ABO group, waiting time, BMI), procedural (e.g., VCA type, UNOS region), and demographic (e.g., age, ethnicity, gender) characteristics, we defined a custom distance function. This function combines $L_{1}$and $L_{2}$norms, depending on whether features are to be assessed jointly—such as spatial distances based on coordinate data—or individually, as in the case of binary indicators. Distances were first computed separately within each domain (demographic, medical, and procedural), normalized to a maximum value of 1, and subsequently combined through a weighted average. Varying the domain weights produced distinct clustering configurations. Table 2 summarizes the weighting schemes applied to generate each cluster solution.

**Supplementary Table 1.** Numerical representations after variable.

| **Variable** | **Representation** |
| --- | --- |
| ABO | Indicator Variables for presence or absence of A Antigens and B Antigens |
| Gender | Indicator for female |
| Ethnicity | 0-1 Encoding for each ethnicity |
| VCA Type | 0-1 Encoding for different types of tissue, function restoration, and region. In addition, we derived a risk profile from a logistic regression on nonsurgical complications (using Hemoglobin A1C, Serum Creatinine, ABO mismatch, ischemia times, and crossmatch results). This yielded a vector representation for each VCA type. |
| UNOS Regions | From cartographic boundary files of the U.S. Census Bureau, we derived one representative coordinate for each UNOS Region. |

**Supplementary Table 2.** Weighting schemes of each cluster configuration.

| **Cluster Configuration** | **Weight Demographic** | **Weight Medical** | **Weight Procedure** |
| --- | --- | --- | --- |
| Demographic-focused | 80% | 10% | 10% |
| Procedure-focused | 10% | 10% | 80% |
| Medical-focused | 10% | 80% | 10% |
| Balanced | 35% | 40% | 25% |
